# A unique wheel-shaped exposed core LSPR-PCF sensor for dual-peak sensing: Applications in the optical communication bands, M-IR region and biosensing

**DOI:** 10.1016/j.heliyon.2024.e33224

**Published:** 2024-06-18

**Authors:** Mohammad Rakibul Islam, Ali Ahnaf Hassan, Shihab Shahriar, Sumaiya Tasnim Adiba, Fahima Shahana Rahman, Safin Zaman, Muhammad Alif Al Hosain

**Affiliations:** Department of Electrical and Electronic Engineering, Islamic University of Technology, Gazipur, 1704, Bangladesh

**Keywords:** Dual peak sensing, Wavelength sensitivity (WS), Amplitude sensitivity (AS), Double peak shift sensitivity (DPSS), Biosensing, Optical communication band

## Abstract

Photonic Crystal Fibers (PCF) effectiveness in practice decreases if the fabrication of the sensor becomes too complex. Keeping this in mind, we propose a one-of-a-kind wheel shaped PCF sensor with an exposed core containing only three air holes with exceptional sensing features. The sensor is equipped with dual plasmonic layers, Indium Tin Oxide (ITO, 10 % wt) and silver (Ag) with a coating of TiO_2_ to enhance the sensing capabilities and provide protection against oxidation. The sensor's distinctive configuration enables it to exhibit two distinct peaks within a range of refractive index from 1.32 to 1.38 for y-polarization and from 1.35 to 1.38 for x-polarization. The sensor specifications have been optimized to achieve the maximum levels of wavelength sensitivity (WS) and double peak shift sensitivity (DPSS). The sensor portrays a WS of 50,652 nm/RIU and the highest DPSS ever recorded, measuring 50,000 nm/RIU. Additionally, the sensor exhibits a significantly high scale of amplitude sensitivity (AS) of 1668.34 RIU^−1^ which is a very remarkable value considering silver as plasmonic material along with an outstanding figure of merit (FOM) of 1017.11 RIU^−1^. In addition, our sensor is able to manifest resolutions in the order of 10^−6^, demonstrating a resolution of 5.94 × 10^−6^ RIU with the deployment of amplitude interrogation method and 1.97 × 10^−6^ RIU with the wavelength interrogation method. The design spans an extensive spectrum, covering ultraviolet to mid-infrared wavelengths, enabling detection of biomolecules and biochemicals, along with operation in the optical communication band.

## 1 Introduction

SPR is a prominent analytical technique used in the investigation of interactivity between biomolecules, such as proteins, DNA, RNA [1], as well as interactions between molecules and surfaces. SPR relies on observing variances in the refractive index adjacent to the metal surface when molecules latch to it [[2], [3], [4], [5]]. Surface Plasmon Resonance with Photonic Crystal Fibers innovatively leverages optical fibers with controlled air hole patterns for enhanced SPR capabilities [6]. SPR sensors have been extensively utilized in the study of protein-protein interactions, DNA and RNA Interactions, bio-sensing and diagnostics, pharmacology, toxicology, nanoparticle interactions, biomedical and biochemical applications and so on [5]. Other mentionable specific fields include applications in THz sensors [[7], [8], [9], [10], [11]], optical communication [12,13], wave transmission [14], detection of blood constituents [15], household oil analysis [16], merger of spectroscopy and RI sensing for transformer oil monitoring [17], heavy metal detection [18], fusion of IMD and EMD based approaches [19], etc.

There are two distinct forms of SPR namely: a) LSPR and b) SPR. The metal-dielectric contact is where the SPR effect predominates, but the nanostructure interface may generally experience LSPR. In SPR sensing [20], initiation of the surface plasmon wave (SPW) typically emerges from the unbound electrons’ vibrations located along the plasmonic layer surface. When a specific wavelength is reached, the alignment connecting the surface plasmon polariton (SPP) mode and the core mode occurs, resulting in the establishment of the phase-matching condition which in turn produces a strong and unique resonance peak. This peak functions as an analyte RI detector since it is very sensitive to even subtle alterations in its RI. Alternatively, when the frequency of a photon colliding with a metal nanoparticle aligns with the resonant frequency of the plasma confined near the surface of the metal particle, the event is called LSPR (Localized Surface Plasmon Resonance). Photon resonance with nanoparticles energizes the plasma, leading to intense light absorption [21,22]. LSPR occurs when metals are exposed to light at the nanoscale level, specifically on nanoparticles as opposed to SPR which occurs on a single substrate, often a thin metal sheet, at the micrometer scale. LSPR sensors, with a shorter electromagnetic field decay length compared to SPR sensors, are gaining prevalence for nanomaterial-sized detecting zones, enhancing sensitivity, reaction time, and throughput within nanostructure confines [23]. Additionally, SPR sensors require more complicated production processes, and their thick cladding layers give them limited mechanical robustness [24].

In addition to the conventional PCF structures [14,[25], [26], [27], [28]], many researchers have also explored the utilization of various irregularly shaped PCF sensors in order to enhance sensing applications and increase the range of measurable quantities, allowing for the development of more cutting-edge technologies. Using Gold as the plasmonic material, Hasan et al. presented a spiral-shaped SPR-PCF sensor with three rings and six arms with birefringence asymmetry [29] which showed a WS of 4600 nm/RIU and an AS of 420.4 RIU^−1^. A rather unique PCF sensor was devised by Jingwei Lv et al. for detecting changes in magnetic fields and temperature of the surrounding area by employing a dual-channel PCF structure where the flat ends are encased in gold films and two silver nanowires are employed along a fan-shaped opening upholding a sensitivity of 31,000 nm/RIU along with a resolution of 3.22 × 10^−6^ RIU in a range of 1.47–1.52 RI [30]. Xiao et al. devised a sensor comprising of an array of Au nanowires deposited over a layer of TiO_2_ which can detect analytes for a wide range of 1.08–1.37 extending from 1210 nm to 2140 nm in terms of wavelength [31].

In addition to structural variations, there have been instances of integrating two plasmonic materials within a single sensor. The integration of TiO_2_/Graphene layers over Gold/Silver-based sensors is primarily to safeguard from oxidation while ensuring proper adhesion [[32], [33], [34], [35]]. The bilayer consisting of TiO_2_/Graphene-Au/Ag also helps to further enhance the sensing parameters. Rifat et al. formed a multi-core flat fiber using a bilayer of TiO_2_ and Au and achieved the topmost result of 23,000 nm/RIU along with an AS of 820 RIU^−1^ [33]. Singh et al. formulated the design of a sensor incorporating a combination of TiO_2_–Au-Graphene and found a remarkable result of 48,900 nm/RIU within a span of 1.32–1.40 RI with an AS of 738.74 RIU^−1^ and FOM of 611.25 RIU^−1^ [35]. Multiple plasmonic materials separately within the same sensor leveraging the dual-resonance phenomenon have also been implemented before [[36], [37], [38], [39]]. In order to reduce costs and simplify fabrication process, the sensors employed a mixture of Au/Ag and Transparent Conductive Oxides (TCO) due to their huge bandgap and high doping density which allows them to display optical characteristics typical of metals in the NIR spectrum [40].

This paper aims to introduce a technologically aligned yet simple sensor design for feasible fabrication, addressing challenges seen in complex multi-air hole PCFs. Unlike intricate designs, our proposed PCF sensor has a straightforward wheel-shaped arrangement with three air holes spaced 120° apart, ensuring ease of fabrication. The sensor demonstrates favorable results in terms of sensing parameters and linearity. The following sections cover design specifications, theoretical simulations, fabrication steps and result discussions.

## 2 Sensor architecture and materials for sensing

**Fig. 1:**
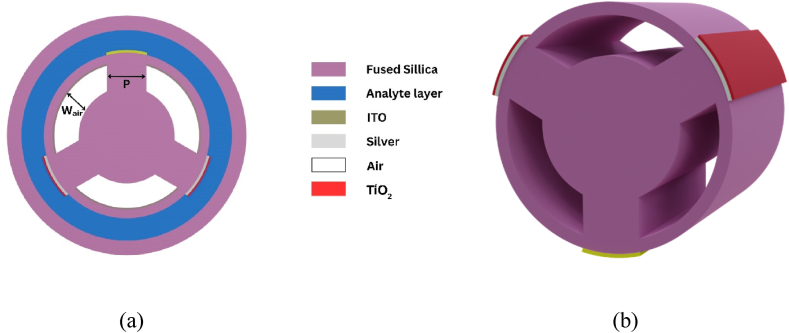
(a) 2-dimensional and (b) 3-dimensional layout of our proposed design.

The 2D and 3D schematic of the sensor we are proposing is displayed in Fig. 1(a) and (b) sequentially. Based on the sensor architecture shown in Fig. 1 (a) and (b), it is clear that the sensor's construction is characterized by a minimalistic design, including only three air holes. In between two neighboring holes, the plasmonic materials are deposited at 120° apart from each other. At the top, a layer of ITO (10 wt%) is used while on the sides, a bimetallic layer of silver and titanium dioxide is placed. ITO is used because of its high carrier concentrations, minimal loss in the infrared region and adjustable photoelectric properties [41]. Liu et al. constructed an ITO-based D-shaped PCF sensor and achieved a WS of 15,000 nm/RIU and an AS of 442.47 RIU^−1^ within a range of 1.22–1.33 [42]. Thus, an ITO layer can contribute to significant changes in resonant wavelength (RW). From the ITO ellipsometry data, we can derive the Drude-Lorentz equation and its parameters using [41]:

(1) $$\varepsilon_{ITO}=\varepsilon_{b}-\frac{{\omega_{p}}^{2}}{\omega \left(\omega +i\gamma_{p}\right)}+\frac{f_{1}{\omega_{1}}^{2}}{\left({\omega_{1}}^{2}-\omega^{2}-i\omega \gamma_{1}\right)}$$

Where $\varepsilon_{ITO}$ denotes the dielectric constant of ITO, $\varepsilon_{b}$ represents the polarization response of core electrons and $\omega_{p}$ is the plasma frequency. The numerical values of the specifications in equation (1) are shown in Table 1. Note that Equation (1) is applicable within the range of wavelength from 350 nm to 2000 nm. Consequently, wavelengths outside this range, which encompass the spectrum responsible for interactions between light and the ITO layer, should not be taken into account. In terms of metals, gold and silver are widely utilized plasmonic materials that have significant appeal in various applications. Silver is preferred over gold in our work because it offers cost-effectiveness, surpasses all other metals in quality factor performance [43], showcasing a significantly more pronounced resonance peak compared to other plasmonic materials. Additionally, it effectively restrains low optical damping and avoids interband transitions, akin to gold [44]. However, silver corrodes and readily oxidizes to generate brittle oxide layers when exposed to aqueous environment [45]. Thus, we coat a layer of TiO_2_ over the Ag layer to eradicate such drawbacks and further improve the performance [46]. Distinctive properties of TiO_2_ at the metal-insulator boundary leads to significant electron accumulation, enhancing the attraction of core-guided evanescent fields toward the exterior. This substantial increase in refractive index not only promotes enhanced interaction between modes but also shifts the sensor's sensitivity towards the near-infrared (NIR) spectrum [47]. Using the Drude-dispersion model, we can calculate silver's dielectric constant which is given as [48]:

(2) $$\varepsilon_{Ag}=1-\frac{\lambda^{2}\lambda_{C}}{{\lambda_{p}}^{2}\left(\lambda_{c}+i\lambda \right)}$$

Here, $\varepsilon_{Ag}$ represents silver's dielectric constant, λ stands for the wavelength in vacuum, $\lambda_{C}$ is the collision wavelength at 17.614 μm, and $\lambda_{p}$, denoting the plasma wavelength, is 0.14541 μm. The relationship between RI and the light wavelength is calculated from the following equation [47]:

(3) $$n_{TiO_{2}}=\sqrt{5.913+\frac{0.2441}{\lambda^{2}-0.0803}}$$

where $n_{TiO_{2}}$ is RI of the TiO_2_ layer and λ denotes wavelength of light. The cladding is filled with fused silica, with the analyte layer on the outside to enable external sensing. The mathematical boundary condition employing the concept of a perfectly matched layer (PML), takes in any amount of radiated energy and keeps it from escaping the computing region, is included in exterior of the analyte layer. The RI profile of the fused silica used in the inner cladding is measured from the Sellmeier equation [2]:

(4) $$n\left(\lambda \right)=\sqrt{1+\frac{B_{1}\lambda }{\lambda^{2}-C_{1}}-\frac{B_{2}\lambda^{2}}{\lambda^{2}-C_{2}}-\frac{B_{3}\lambda^{2}}{\lambda^{2}-C_{3}}}$$

Where silica's RI is presented by n(λ) and the wavelength in μm is λ and the values of the other constants are mentioned in Table 1.

**Table 1:** Sellmeier Constants & Drude-Lorentz Parameters of ITO (10 wt%).

Sellmeier Constants (Fused Silica)		Drude-Lorentz Parameters of ITO (10 % wt)	
B_1_	0.696	$\varepsilon_{b}$	3.528
B_2_	0.408	$\omega_{p}\left[eV\right]$	1.78
B_3_	0.897	$\gamma_{p}\left[eV\right]$	0.155
C_1_ (μm^2^)	0.0047	$f_{1}$	0.3884
C_2_ (μm^2^)	0.014	$\omega_{1}\left[eV\right]$	4.210
C_3_ (μm^2^)	97.934	$\gamma_{1}\left[eV\right]$	0.0919

**Table 2:** Sensor performance parameters after optimization.

Parameter Name	Initial Width (nm)	Final Width (nm)	DPSS after Optimization (nm/RIU)	WS after Optimization (nm/RIU)
$t_{ITO}$	30	15	3010.5	3566
$t_{Ag}$	30	45	3824	4379
$t_{TiO_{2}}$	5	35	15184.5	15741
$w_{air}$	2000	2000	15184.5	15741
p	2000	1600	20961	21498.5

The combination of the plasmonic materials along with the unique structural configuration of our sensor provides novelty to our work. In light of the currently available literature, research comprising the blend of ITO and Ag/TiO_2_ bimetallic layer within the same sensor is yet to be explored. Initially, thickness of the ITO layer, $t_{ITO}$ and Ag layer, $t_{Ag}$ were kept at 30 nm, TiO_2_ layer ($t_{TiO_{2}}$) at 5 nm, width of the air hole, $w_{air}$ at 2 μm and pitch, p at 2 μm. The diameter of the optical fiber core was kept at 5 μm, the width of the analyte and PML layer being 2 μm and 1 μm respectively. The numerical examination is conducted by applying the Finite Element Method (FEM) of the software COMSOL Multiphysics 5.6.

**Fig. 2:**
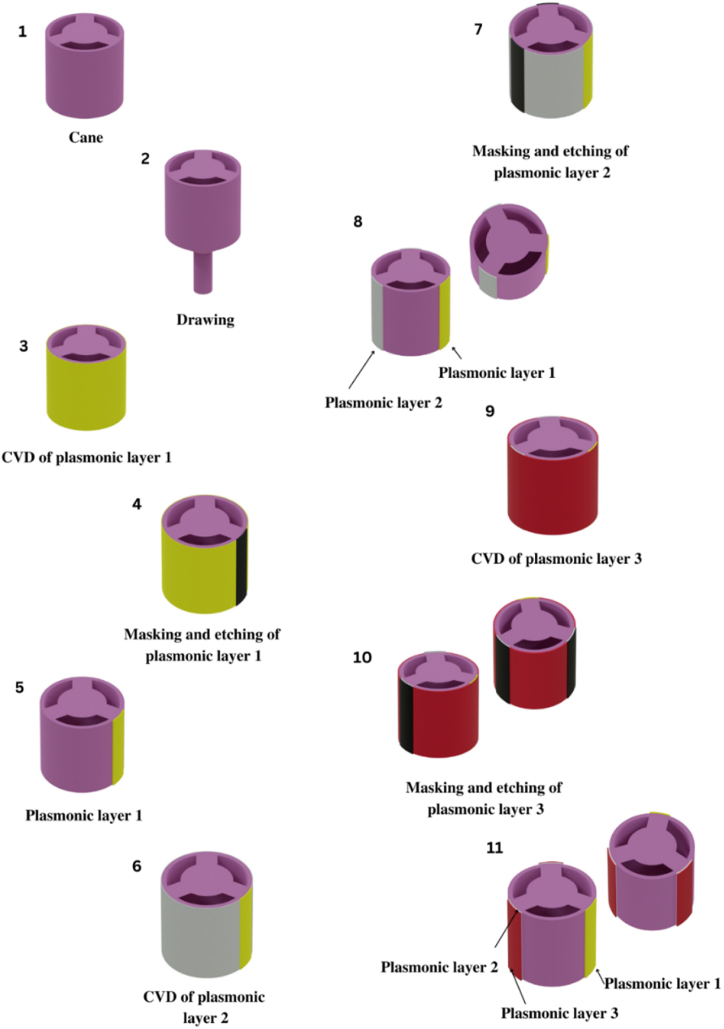
Potential fabrication steps.

The manufacturing techniques currently employed for producing PCF structures can also be applied to fabricate our sensor. The possible steps needed to deploy our sensor practically are illustrated in detail in Fig. 2. Our distinctive PCF design features a wheel-like structure incorporating three air holes, each of which assumes an arc-like shape. These shapes are possible to be crafted harnessing the widely used stack and draw method, a common approach in PCF fabrication [49]. The process involves creating glass tubes with the desired arc-like air hole patterns, stacking them, and then drawing them into a fiber while preserving the designated geometry. The CNC drilling machine can also be used to define the sensor's framework, which can be used to drill out the exact geometries. The incorporation of plasmonic layers can be achieved through the utilization of a double step photolithography method [50]. The first step involves the application of a plasmonic layer onto the fiber's exterior applying the chemical vapor deposition (CVD) process [51]. Next, the attainment of the first layer can be achieved through the utilization of a mask to selectively cover the designated region. Using this method, the ITO, silver and TiO_2_ layers can be coated around the drawn cane. The plasmonic layers possess dimensions that facilitate their seamless integration into the PCF by the CVD technique. Additionally, the air holes are sufficiently large to guarantee effective incorporation.

## 3 Dispersion features of the sensor

The electron frequency alignment in the plasmonic material of the sensor should match the frequency of the evanescent field. To ensure optimal light confinement within the fiber, it is vital to achieve resonance by synchronizing the effective indices of both modes at a designated wavelength. Simultaneously, during this state of resonance, there may be an observable rise in peak loss due to confinement.

**Fig. 3:**
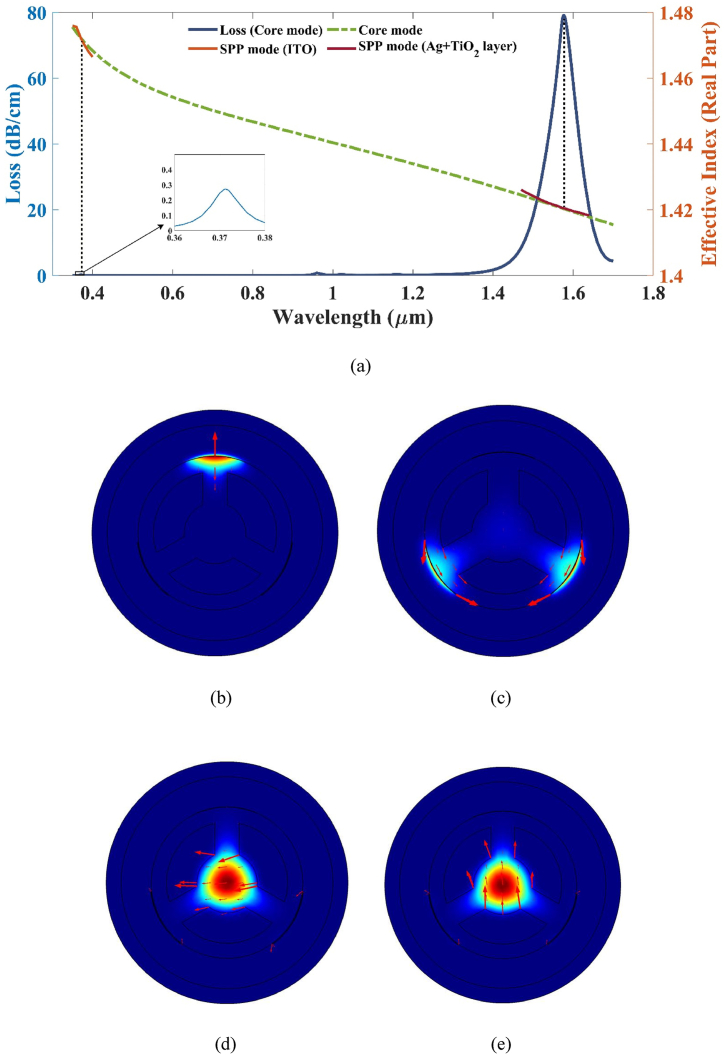
(a) Dispersion relationship of the double peak displayed with the CL (b) SPP along ITO layer (c) SPP mode along Ag + TiO_2_ layer (d) x-pol (e) y-pol for an analyte RI of 1.37.

The sensor is composed of a thin layer of ITO coupled with two separate bimetallic layers of Ag and TiO_2_. This combination leads to the creation of an evanescent field which separates into two parts, leading to the existence of two discernible SPP modes. However, the interaction between the two modes occurs throughout a broad spectrum of frequencies, owing to the distinct properties exhibited by silver and ITO. Consequently, two separate peaks emerge along with phase-matching conditions, thereby hindering the occurrence of distinct resonance wavelengths for both polarization states. Fig. 3(b) and (c) reveal that the SPP mode manifests alongside the individual plasmonic layers at distinct wavelengths, as illustrated in the dispersion relationship in Fig. 3(a). The corresponding core mode light confinement in the sensor is portrayed in Fig. 3(d) and (e). This observation validates the assertion that the sensor's first peak corresponds to the presence of the ITO layer, while the second peak originates from the layer comprised of the silver and titanium dioxide bimetallic configuration.

## 4 Optimization of sensor specifications using W-DPS method

To get the best out of the sensor's potential, we need to properly optimize the sensor specifications. In this work, we propose the simultaneous use of the wavelength interrogation method as well as the double peak shift interrogation method (W-DPS interrogation method). The WS of a sensor is evaluated in the following way [52]-

(5) $$S_{\lambda }=\Delta \lambda_{peak}/\Delta n_{a}\left(nm/RIU\right)$$

Where $S_{\lambda }$ denotes the wavelength sensitivity, $\Delta \lambda_{peak}$ is the displacement of two consecutive RW and $\Delta n_{a}$ depicts the variation in RI. The confinement loss (CL) spectra are measured with the equation [52]-

(6) $$\alpha \left(dB/cm\right)=8.686\times \left(\frac{2\pi }{\lambda }\right)\times \text{Im}\left(n_{eff}\right)\times 10^{4}$$

On the other hand, the double peak shift sensitivity or DPSS, a special variant of the WS parameter that is used when the sensor is specially designed using two separate plasmonic materials, is formulated using the following equation [36]-

(7) $$S_{p-p}=\frac{{\left(\lambda_{p2}-\lambda_{p1}\right)}_{n_{b}}-{\left(\lambda_{p2}-\lambda_{p1}\right)}_{n_{a}}}{n_{b}-n_{a}}\times 10^{3}\left(nm/RIU\right)$$

where, $S_{p-p}$ represents the DPSS at $n_{a}$ RI, $\lambda_{p1,n_{b}}$ and $\lambda_{p1,n_{a}}$ signifies the wavelengths of the 1st peaks, and $\lambda_{p2,n_{b}}$ and $\lambda_{p2,n_{a}}$ depicts the RWs of the 2nd peaks at RI $n_{b}$ and $n_{a}$, respectively. Sensor parameters are chosen in such a way that both the WS and DPSS are maximized.

The optimization was carried out for an RI of 1.36 and 1.37 along the y-polarization mode. Based on the initial set of values as mentioned in section-II, a DPSS of 2397 nm/RIU was found and the consequent values of WS for the two peaks found were 1172 nm/RIU and 3569 nm/RIU.

### 4.1 ITO layer width ($t_{ITO}$)

At first, the ITO layer was optimized. Since our goal is to maximize the DPSS of the whole system, we need to maximize the separation among the two peaks. Increasing the dimension of the ITO layer would result in a shift of the 1st peak towards higher wavelengths, thereby leading to a reduction in the DPSS of the system. So, the depth of the ITO layer was reduced to increase the DPSS of the system. From 30 nm, the thickness was decreased with an interval of 5 nm. At 15 nm, the RW responsible for ITO layer was found at 367.275 μm at RI = 1.36. If it was decreased any further, the RW would shift before the 350 nm benchmark. Equation (1) is valid only for a range of 350 nm–2000 nm. Thus, the optimum thickness was selected at 15 nm.

**Fig. 4:**
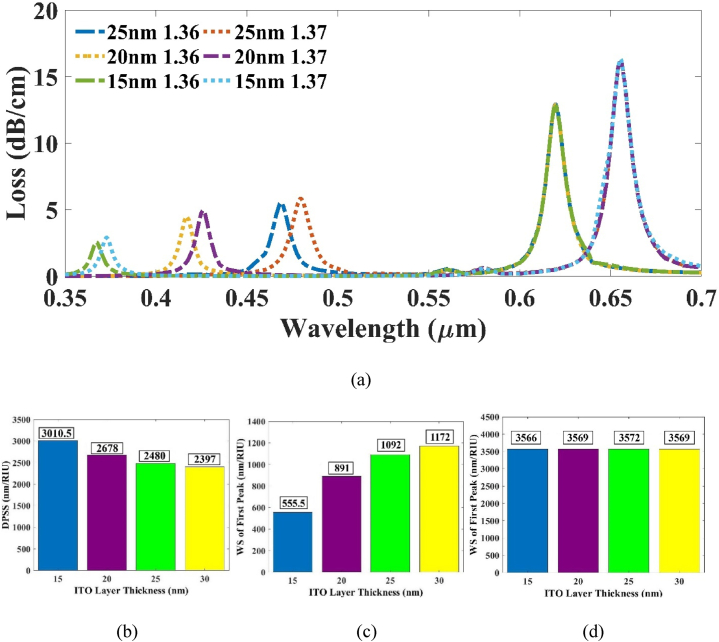
(a) CL vs wavelength plot for varying $t_{ITO}$ at RI 1.36 & 1.37 (b) Effect of DPSS with $t_{ITO}$ (c) WS of 1st peak and (d) 2nd peak respectively.

From Fig. 4 (c), we can see the trend of decrease of WS of 1st peak with the decrease in the width of ITO layer which consequently resulted in the increase of DPSS as shown in Fig. 4 (b). The WS of 2nd peak also has minimal change due to this variation as shown in Fig. 4 (d). Furthermore, from Fig. 4 (a), the CL of the 1st peak was also decreasing with the decrease in the thickness. The DPSS value after optimizing the ITO layer was found at 3010.5 nm/RIU. Due to this variation in $t_{ITO}$, the detection range increased from 367.275 nm to 619.755 nm wavelength.

### 4.2 Ag layer width ($t_{Ag}$)

**Fig. 5:**
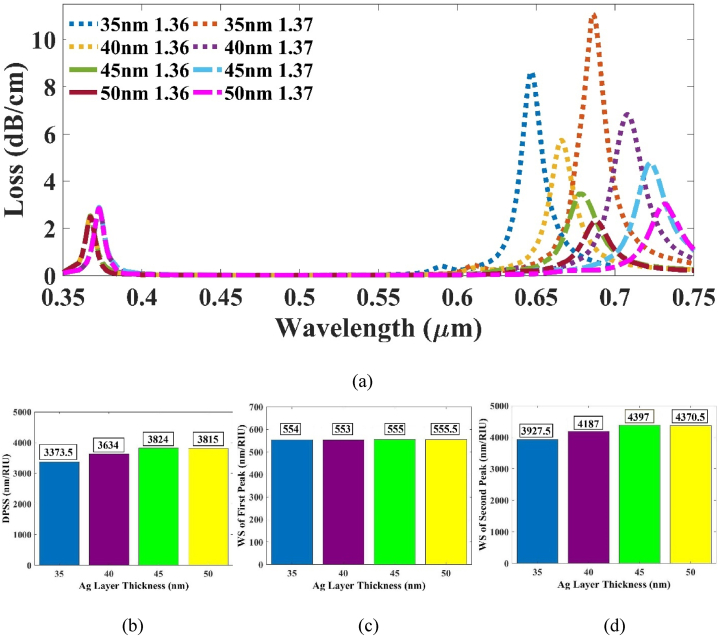
(a) CL vs wavelength plot for varying $t_{Ag}$ at RI 1.36 & 1.37 (b) Effect of DPSS with varying $t_{Ag}$ (c) WS of 1st peak and (d) 2nd peak respectively.

Secondly, the Ag layer was optimized. If the Ag film width is increased, then the RW will experience a right shift. Our motive is to find the thickness for which the WS of the 2nd peak is the highest, which in turn will make the DPSS highest. With this in mind, we increased the dimension of the Ag layer from 30 nm to 50 nm. The WS of the 2nd peak increased until 45 nm width and reached a value of 4379 nm/RIU [Fig. 5(b)] and the DPSS was found 3824 nm/RIU [Fig. 5(d)]. At 50 nm, WS decreased to 4370.5 and the DPSS attained was 3815 nm/RIU. Thus, the optimized value of Ag layer was picked at 45 nm.

We can see that the 1st peak has negligible effect on the variation of $t_{Ag}$ value from Fig. 5 (c). The detection range increased to 678.71 nm due to the increase in $t_{Ag}$ which can be seen from the CL vs wavelength plot in Fig. 5 (a).

### 4.3 TiO_2_ film width ($t_{TiO_{2}}$)

**Fig. 6:**
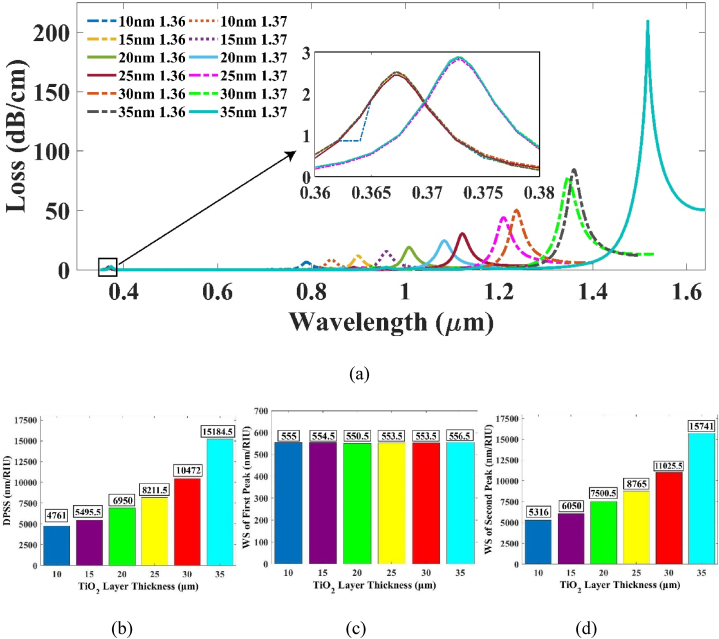
(a) CL vs wavelength plot for varying $t_{TiO_{2}}$ at RI 1.36 and 1.37 (b) Effect of DPSS with varying $t_{TiO_{2}}$ (c) WS of 1st peak and (d) 2nd peak respectively.

Initially, the TiO_2_ film was deposited as a measure of protection from oxidation of the silver layer. However, the TiO_2_ film can also contribute to a notable enhancement in the system's sensitivity if we consider the bimetallic layer as a whole [46]. The value of $t_{TiO_{2}}$ was increased from the previously set 5 nm value at a step of 5 nm. It was discovered that the DPSS and WS of the 2nd peak was showing significant rise with the increment of $t_{TiO_{2}}$ value as shown respectively in Fig. 6 (b) and (d). At a width of 35 nm, the combined silver and TiO_2_ bimetallic layer reached a total thickness of 80 nm, which introduced challenges in achieving effective coupling between the SPP and the core mode. Further expanding the $t_{TiO_{2}}$ width exacerbated the difficulty of meeting the phase matching condition, leading to the point where the resonance condition could no longer be sustained. Consequently, the final thickness of the TiO2 film was selected as 35 nm. Since the TiO_2_ layer was deposited over the Ag layer, the variation in its thickness did not affect the WS of 1st peak [Fig. 6(c)].

In Fig. 6(a), the spectrum extends into the optical communication band. At an RI of 1.36, the RW occurs at 1395.77 nm, and at an RI of 1.37, it shifts to 1517.18 nm. Consequently, the sensor covers both the E-band and S-band within the optical communication range.

### 4.4 Air hole width ($w_{air}$) and pitch (p)

The width of the air holes was adjusted by both increasing and decreasing with increments of 100 nm. When increased by 100 nm, the DPSS decreased to 10865.5 nm/RIU, and the WS of the 1st and 2nd peaks decreased to 508.5 nm/RIU and 11374 nm/RIU, respectively. Conversely, decreasing the width resulted in an excessive gap separating the air holes and the plasmonic layers, leading to difficulties in achieving phase matching condition. Consequently, the resonant state could not be fulfilled in this scenario as well. As a result, the optimal value for the $w_{air}$ was determined to be the pre-set 2 μm.

**Fig. 7:**
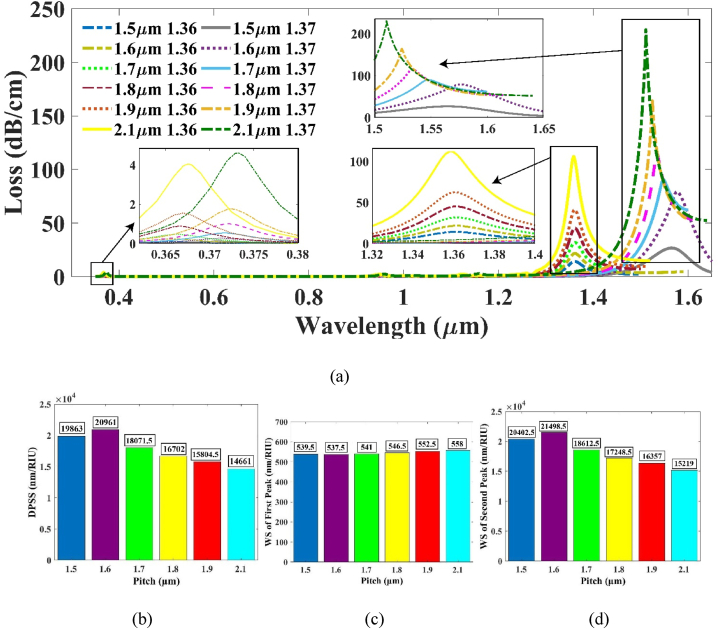
(a) CL vs wavelength plot for varying pitch at RI 1.36 & 1.37 (b) Effect of DPSS with varying pitch (c) WS of 1st peak and (d) WS of 2nd peak respectively.

Finally, the pitch, i.e., the spatial distance of two adjacent air holes, was adjusted to get the optimal values of the WS of the 2nd peak and DPSS. Upon increasing the pitch, the CL was amplified, resulting in a reduced separation between the resonance wavelengths (RWs). Consequently, both the DPSS and WS decreased for the sensor. In contrast, decreasing the pitch brought the air holes closer together, causing a reduction in CL seen from Fig. 7 (a). The trend of the DPSS and WS increasing as the pitch decreased is depicted in Fig. 7(b), (c), and (d). At a pitch value of 1.6 μm, the highest shift was observed. The resonant wavelengths were measured at 1361.495 nm and 1576.48 nm for RI of 1.36 and 1.37, correspondingly. This expansion of the sensor's application extends into the L-Band of the optical spectrum.

A reduction in the pitch led to a significant decrease in the coupling length (CL). At an RI of 1.36, the 1st peak manifested with minimal loss, measuring only 0.238 dB/cm, while the 2nd peak exhibited a loss of 21.7326 dB/cm. This observation underscores the fact that our suggested sensor not solely attains a high level of sensitivity, additionally also lends itself well to the fabrication process, showcasing remarkably low loss characteristics. The sensor's final optimized DPSS was established to be 20961 nm/RIU while the WS of the 2nd peak was as high as 21498.5 nm/RIU.

Previous research in this area suggests that the sensor's sensing characteristics are mostly unaffected by the analyte and PML layer's optimization. There is no change in the sensor's WS, and the AS fluctuates just a little. As the optimization was based on a combined effort of WS and DPSS, the analyte and PML layer was unaltered. The final sensor configuration and result after optimization is shown in Table 2.

## 5 Sensor **performance** traits

### 5.1 Double peak shift sensitivity

**Fig. 8:**
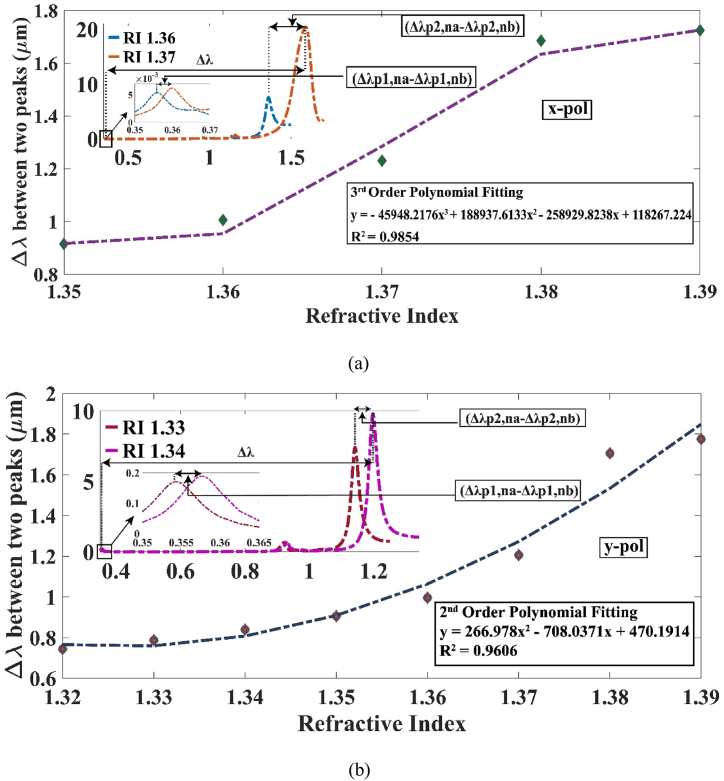
Difference of RW along the (a) x polarization and (b) y polarization.

The unique sensor we modelled is capable of depicting dual peaks for a range of RI 1.35–1.38 along x-polarization and 1.32–1.38 RI along y-polarization. The trend of separation of the two resonant peaks with change in RI is portrayed clearly in Fig. 8 (a) and (b) for x-pol and y-pol respectively. Utilizing equation (7), the sensor demonstrated a remarkable DPSS of 50,000 nm/RIU across the y-pol at an RI of 1.37. Similarly, the highest DPSS observed along the x-polarization was 45,503 nm/RIU. These DPSS values significantly surpass the previous highest recorded value of 27,341.5 nm/RIU [37]. Consequently, our sensor establishes a new benchmark for the highest DPSS achieved to date. It's noteworthy that we didn't analyze the sensor for x-pol before an RI of 1.35 or for y-pol before an RI of 1.32. This is attributed to the resonance condition of the ITO layer exceeding the 350 nm limit set by equation (1) [41].

**Fig. 9:**
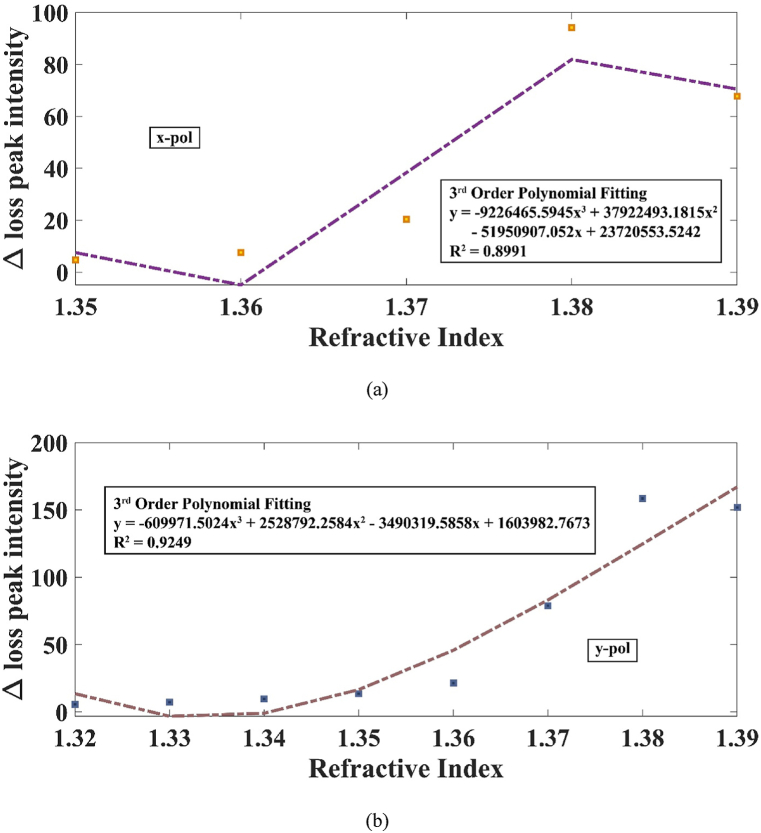
Relation of RI with the difference of the loss peak intensity across (a) x-polarization and (b) y-polarization.

**Fig. 10:**
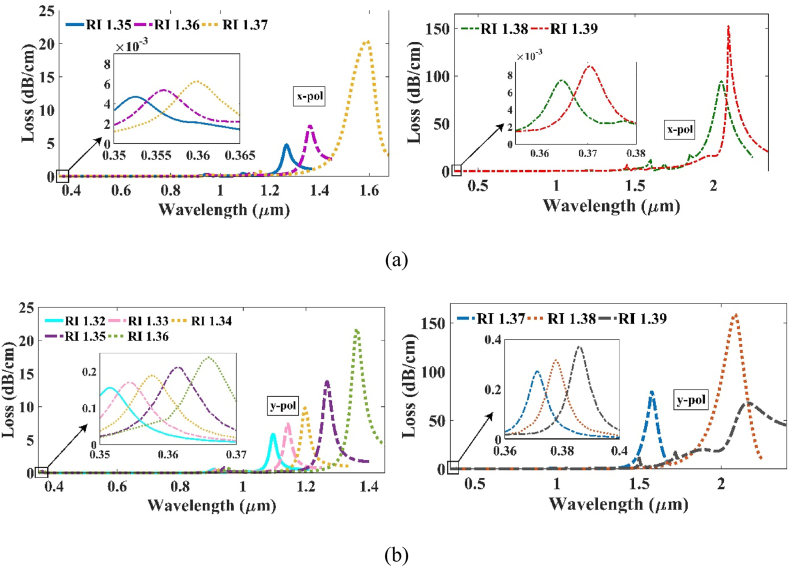
CL vs wavelength along (a) x-pol at a range of 1.35–1.39 and (b) 1.32–1.39 RI for y-pol.

The transfer of energy from the two modes exhibits wavelength-dependent variations due to the discrepancy in loss strength among both the resonance peaks, which can be credited to light scattering phenomena [36]. Fig. 9 (a) and (b) clearly indicate that the x-polarization mode exhibits lower losses compared to its y-polarization counterpart. In particular, the x-polarization mode shows the highest loss disparity between the two peaks at 94.2346 dB/cm for 1.38 RI. On the contrary, the y-polarization mode demonstrates a loss difference of 158.4287 dB/cm at the same RI. Fig. 8, Fig. 10 explain that the shift in RW decreased after RI 1.38. Thus, the investigation was stopped after 1.38 for both polarizations. The sensor's range extends from 350 nm to 2094.79 nm for x-pol and 2160.88 nm for y-pol. Thus, the sensor's application extends from the ultra-violet spectrum to even Mid-Infrared (M-IR) region.

### 5.2 **wavelength** sensitivity

Using equation (5), the WS of the sensor was examined for both the polarizations. Here, we will consider only the 2nd peak for measuring the WS as the purpose of the 1st peak was to increase the difference between the two peaks within the same refractive index. Our sensor can withhold a highest WS of 45,978.5 nm/RIU along x-pol and 50,652 nm/RIU along y-pol for a refractive index of 1.37. This indicates that the sensor can precisely detect analytes owing to the high shift in resonant wavelength. For the RI 1.38, the WS drastically drops to 4500 nm/RIU for x-pol and 7788 nm/RIU for y-pol. For this reason, the investigation was not continued further. The loss curve for the detection range along both the x and y-polarizations are displayed in Fig. 10 (a) and (b) respectively.

### 5.3 Amplitude sensitivity

The Amplitude Sensitivity (AS) is a term utilized to quantify the sensitivity by considering the attenuation at a specific light wavelength. The measurement is determined through the utilization of the subsequent equation [31]:

(8) $$S_{A}=-\frac{1}{\alpha \left(\lambda ,n_{a}\right)}\frac{\partial_{a}\left(\lambda ,n_{a}\right)}{\partial n_{a}}\left(RIU^{-1}\right)$$

**Fig. 11:**
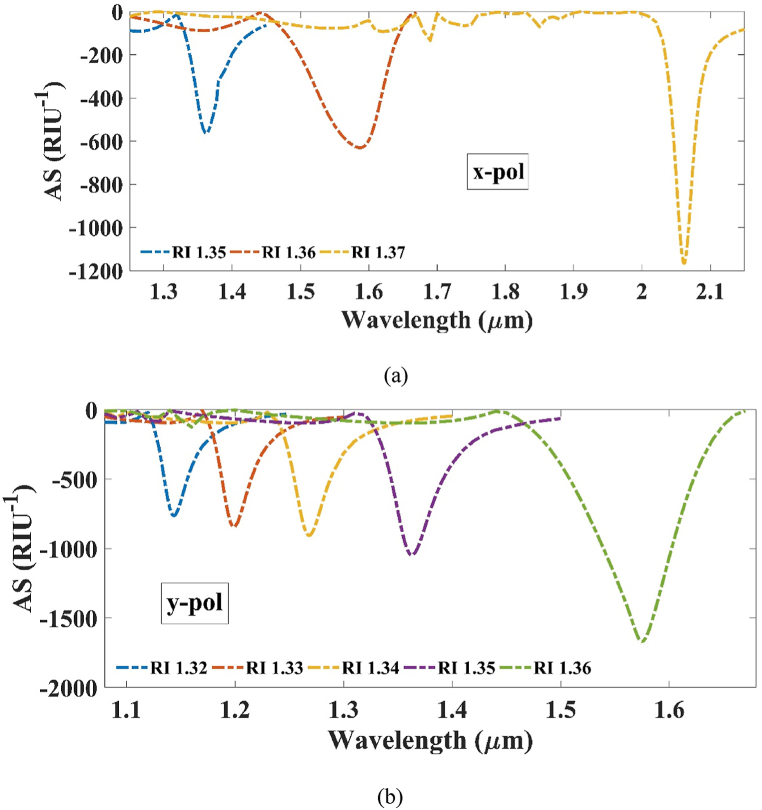
Amplitude sensitivity for (a) x-polarization and (b) y-polarization.

This method is relatively cheaper as it does not require the interaction of light. Our proffered sensor exhibits a highest AS of 1166.7435 RIU^−1^ for x-pol at RI 1.37 and for y-pol, it was found to be 1668.34 RIU^−1^ at RI 1.36 as can be viewed from Fig. 11 (a) and (b) respectively. After the mentioned refractive indices, the AS starts to degrade and thus the parameter was not further measured.

### 5.4 Sensor resolution

The sensor resolution is an important criterion to take note of in order to precisely detect analytes for minute alterations in the analyte's RI. It can be computed for both amplitude and wavelength sensitivity measuring methods which is shown in equations (9), (10) [36]:

(9) $$R_{A}=\frac{\Delta n_{A}}{S_{A}}\left(RIU\right)$$

(10) $$R\left(w\right)=\frac{\Delta \lambda_{\min}}{S_{\lambda }}\left(RIU\right)$$

Here, $R_{A}$ = Amplitude Resolution in RIU, $\Delta n_{A}$ = 0.01, $S_{A}$ = AS in RIU^−1^, R(w) = Wavelength Resolution in RIU, $\Delta \lambda_{\min}$ = 0.1 nm and $S_{\lambda }$ = Wavelength Sensitivity in nm/RIU. Along x-pol, the amplitude resolution was 8.571 × 10^−6^ RIU while along y-pol, it was 5.994 × 10^−6^ RIU. In case of wavelength resolution, x-pol showed the highest result of 2.175 × 10^−6^ RIU and 1.974 × 10^−6^ RIU for y-pol. Therefore, our sensor exhibits the capability to detect subtle variations on the scale of 10^−6^.

### 5.5 Linearity

**Fig. 12:**
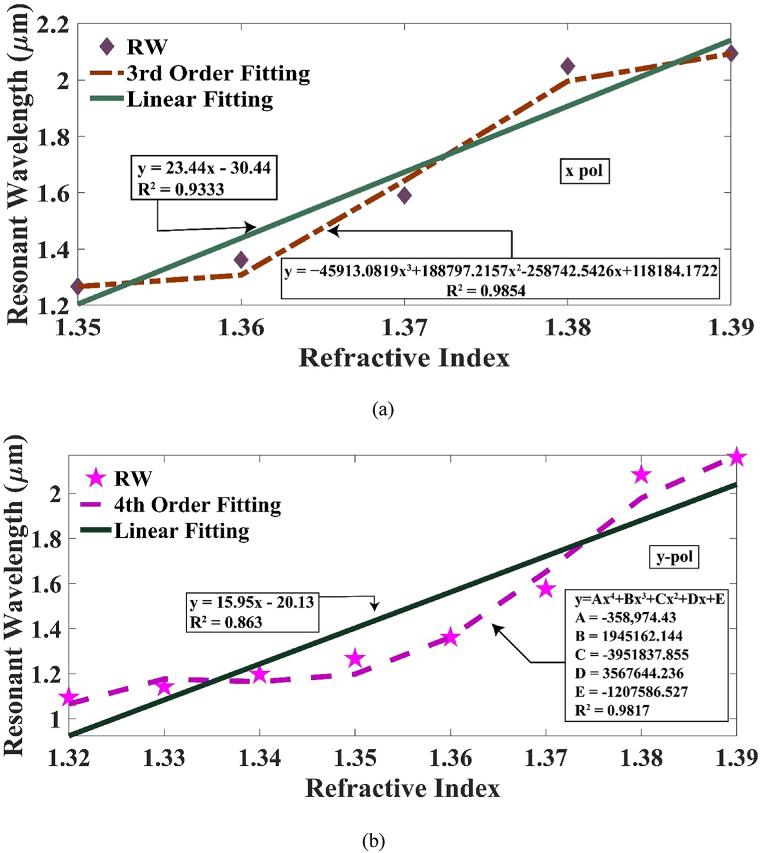
Polynomial modelling for the Ag peak across (a) x-polarization and (b) y-polarization.

A strong correlation between the RI of the material being analyzed and the RW suggests the presence of an exceptional sensor that might be conveniently calibrated. The plot of RW with refractive index along x-pol follows an equation of y = 23.44x – 30.44 with a R^2^ = 0.9333. A third order polynomial equation, when applied to the scatter plot, yields an R^2^ value of 0.9854. For y-pol, the linear fitting equation yields an R^2^ estimation of 0.863 with y = 15.95x – 20.13 but if the order is increased to the fourth degree, then the R^2^ value increases to 0.9817. This analysis proves the high quality of our sensor. This is upheld in Fig. 12 (a) and (b).

### 5.6 Figure of merit

The equation below is used to formulate the Figure of Merit (FOM) [52].:

(11) $$FOM=\frac{S_{\lambda }}{FWHM}\left(RIU^{-1}\right)$$

where FWHM denotes the Full Width at Half Maximum. Across y-pol, our sensor demonstrates a high FOM of 1017.108 RIU^−1^ and 421.05 RIU ^−1^ in case of the other polarization. The FOM along the y-polarization is notably high, a characteristic not commonly observed in silver-based sensors.

### 5.7 Applications in the optical communication band & M-IR region

The term “optical communication band” is used to describe a specific region of the electromagnetic spectrum that is typically employed for the transmission of data through light signals. The spectral band encompasses a spectral range of 1260 nm–1650 nm. This band is selected because it is where optical fibers experience the least attenuation from processes like light absorption and scattering. Because of this, high-speed, effective data transfer with little signal degradation is possible within this wavelength domain [53]. These range can be broken down into the following categories:

• O-Band: 1260 nm–1360 nm
• E-Band: 1360 nm–1460 nm
• S-Band: 1460 nm–1530 nm
• C-Band: 1530 nm–1565 nm
• U-Band: 300 nm–400 nm

We can see the resonant wavelength of the 2nd peak of our sensor occurs within a span of 1095 nm–2160 nm which covers the optical communication band as well as the mid-infrared (M-IR) band range (see Table 3a, Table 3b). Thus, our sensor can be employed in applications like optical amplifiers, fiber-optic communications, sensing systems, telecommunications, etc. [54]. Applications in the M-IR region include spectroscopy, gas sensing, medical imaging, remote sensing, environmental monitoring, etc. [55,56].

**Table 3a:** Performance traits of the sensor at all investigated RI (X-Pol).

RI	RW (nm)	WS (nm/RIU)	AS (RIU^−1^)	DPSS (nm/RIU)	Wavelength Resolution (RIU) ( × 10^−6^)	Amplitude Resolution (RIU) ( × 10^−6^)	FOM (RIU^−1^)
1.35	1266.98	9451.5	563.18	9115	10.58	17.756	203.7
1.36	1361.49	22851.5	630.47	22454.5	4.376	15.86	382.07
1.37	1590.01	45978.5	1166.74	45503	2.175	8.57	421.05
1.38	2049.79	4499.5	–	3921.5	22.25	–	31.687

**Table 3b:** Performance traits of the sensor at all investigated RI (Y-Pol).

RI	RW (nm)	WS (nm/RIU)	AS (RIU^−1^)	DPSS (nm/RIU)	Wavelength Resolution (RIU) ( × 10^−6^)	Amplitude Resolution (RIU) ( × 10^−6^)	FOM (RIU^−1^)
1.32	1095.16	4621.5	762.478	4336	21.64	13.115	163.94
1.33	7399.99	5548.5	844.177	5218.5	18.02	11.846	180.733
1.34	1196.86	6985	905.161	6602	14.32	11.048	204.24
1.35	1266.71	9478.5	1046.81	9031.5	10.55	9.553	236.667
1.36	1361.49	21499	1668.34	20961	4.65	5.994	431.697
1.37	1576.48	50652	–	50000	1.97	–	1017.11
1.38	2083	7788	–	6976	12.84	–	46.083

### 5.8 Opportunities as a biosensor

Our sensor exhibits considerable potential to be a biosensor, attributed to its capacity to establish resonance conditions across a broad spectrum. Specifically, the sensor can achieve phase matching conditions within an extent of 350 nm–2083 nm along the x-polarization, and up to 2160 nm along the y-polarization. These extended wavelength ranges offer distinctive attributes suitable for the detection of biomolecules and biochemicals. Various applications of this capability are outlined in Table 4:

**Table 4:** List of Some Biomolecules and Biochemicals within the Range of our Sensor.

Analyte	Analyte RI	DPSS (nm/RIU)	WS (nm/RIU)	AS (RIU^−1^)
Methyl, Water	1.33	5218.5 (y-pol)	5548.5 (y-pol)	844.17 (y-pol)
Milk, Plasma	1.35	9115 (x-pol)	9478.5 (y-pol)	1046.81 (y-pol)
WBC, Basal cells, Acetone	1.36	22454.5 (x-pol)	22851.5 (x-pol)	1668.34 (y-pol)
Acetic Acid	1.37	50000 (y-pol)	50652 (y-pol)	1166.74 (x-pol)
Skin Cell	1.38	6976 (y-pol)	7788 (x-pol)	–

**Table 5:** Comparative assessment in the context of relevant studies.

Ref.	Materials	RI range	AS (RIU^−1^)	WS (nm/RIU)	WR (RIU)	AR (RIU)	FOM (RIU^−1^)	DPSS (nm/RIU)
[42]	ITO	1.22–1.33	442.47	15,000	–	6.67*10^−6^	–	–
[57]	ITO + ZnO	1.30–1.38	–	10,000	2.0 × 10^−5^	–	–	–
[58]	Ag/Graphene	1.46–1.49	418	3,000	3.33 × 10^−5^	2.4*10^−5^	–	–
[59]	Ag/Graphene	1.33–1.35	72.47	2,520	3.97 × 10^−5^		–	–
[60]	Ag/Graphene	1.33–1.37	216	3,700	2.7 × 10^−5^	4.6*10^−5^	–	–
[61]	Ag/Graphene/TiO_2_	1.32–1.38	–	8,750	–	–	346.5	–
[62]	Ag/TiO_2_	1.33–1.40	2245	10,300	9.71 × 10^−6^	4.45*10^−6^	480	–
[36]	AZO + Au	1.27–1.42	8485.2	46,300	2.16 × 10^−6^	1.18*10^−6^	–	16,500
[38]	GZO + Au	1.30–1.40		11,480	8.71 × 10^−6^		–	11,720
[39]	AZO + GZO	1.30–1.41	11609.67	11,088.5	9.874 × 10^−6^	8.61*10^−7^	1558.8	10,890.35
[37]	GZO + Ag	1.27–1.41	875.72	27,360	1.142 × 10^−5^	3.65*10^−6^	243.4	27,341.5
Our Work	ITO+ Ag/TiO_2_	1.32-1.38	1668.34	50,652	1.97 × 10^−6^	5.99 × 10^−6^	1017.1	50,000

## 6 Comparative analysis with related works

Based on Tables 5 and it becomes apparent that our sensor embodies an optimal blend of exceptional sensing parameters. Furthermore, it achieves the highest recorded value of DPSS to date, thus attesting to the excellence of our sensor.

## 7 Conclusion

In conclusion, our unique sensor of only three air holes making a wheel shaped structure holds remarkable potential in the field of biosensing and optical communication. The sensor's capacity to detect biomolecules and biochemicals is facilitated by its ability to cover an extensive array of spectrum, which is attributed to the unique qualities of light within that specific wavelength spectrum. The sensor exhibits promising applications in the realm of telecommunications as well. The sensor's distinctive structural arrangement grants it the capability to have a DPSS of 50,000 nm/RIU which is sufficiently higher than the previously documented values. It can also demonstrate a WS of 50,652 nm/RIU and an AS of 1668.34 RIU^−1^ with a supreme FOM of 1017.11 RIU^−1^. The sensor exhibits a high level of precision in spotting alterations in analyte's RI, given that it offers an accuracy level on the magnitude of 10^−6^ employing both amplitude and wavelength interrogation methods. The PCF sensor is investigated in an RI range of 1.35–1.38 along x-polarization and 1.32–1.38 along y-polarization. The ease of fabrication along with the high-quality sensing performance allows the sensor to be practically implemented while ensuring the potential applications in the field of telecommunications and medical engineering.

## Funding statement

This study did not receive dedicated funding from any public, commercial, or non-profit sources.

## Data availability statement

No data was used for the research described in the article.

## CRediT authorship contribution statement

**Mohammad Rakibul Islam:** Writing – review & editing, Supervision, Project administration, Conceptualization. **Ali Ahnaf Hassan:** Writing – review & editing, Writing – original draft, Validation, Software, Methodology, Investigation, Formal analysis, Conceptualization. **Shihab Shahriar:** Writing – review & editing, Writing – original draft, Validation, Software. **Sumaiya Tasnim Adiba:** Writing – review & editing, Writing – original draft, Validation, Software. **Fahima Shahana Rahman:** Writing – review & editing, Writing – original draft, Validation, Software. **Safin Zaman:** Writing – review & editing, Writing – original draft, Investigation. **Muhammad Alif Al Hosain:** Writing – review & editing, Writing – original draft, Investigation.

## Declaration of competing interest

The authors declare that they have no known competing financial interests or personal relationships that could have appeared to influence the work reported in this paper.
